# A Novel Heterozygous Missense Mutation in* GNAT1* Leads to Autosomal Dominant Riggs Type of Congenital Stationary Night Blindness

**DOI:** 10.1155/2018/7694801

**Published:** 2018-04-23

**Authors:** Christina Zeitz, Cécile Méjécase, Mathilde Stévenard, Christelle Michiels, Isabelle Audo, Michael F. Marmor

**Affiliations:** ^1^Sorbonne Université, INSERM, CNRS, Institut de la Vision, Paris, France; ^2^CHNO des Quinze-Vingts, DHU Sight Restore, INSERM-DGOS CIC 1423, Paris, France; ^3^Institute of Ophthalmology, University College of London, London, UK; ^4^Department of Ophthalmology and Byers Eye Institute, Stanford University School of Medicine, Palo Alto, CA, USA

## Abstract

Autosomal dominant congenital stationary night blindness (adCSNB) is rare and results from altered phototransduction giving a Riggs type of electroretinogram (ERG) with loss of the rod a-wave and small b-waves. These patients usually have normal vision in light. Only few mutations in genes coding for proteins of the phototransduction cascade lead to this condition; most of these gene defects cause progressive rod-cone dystrophy. Mutation analysis of an adCSNB family with a Riggs-type ERG revealed a novel variant (c.155T>A p.Ile52Asn) in* GNAT1* coding for the *α*-subunit of transducin, cosegregating with the phenotype. Domain predictions and 3D-modelling suggest that the variant does not affect the GTP-binding site as other* GNAT1* adCSNB mutations do. It affects a predicted nuclear localization signal and a part of the first *α*-helix, which is distant from the GTP-binding site. The subcellular protein localization of this and other mutant GNAT1 proteins implicated in CSNB are unaltered in mammalian GNAT1 overexpressing cells. Our findings add a third* GNAT1 *mutation causing adCSNB and suggest that different pathogenic mechanisms may cause this condition.

## 1. Introduction

Congenital stationary night blindness (CSNB) is a clinically and genetically heterogeneous nonprogressive retinal disease, characterized by absent or severely reduced vision in the dark, diminished rod function, and variably other abnormalities such as mild visual loss, nystagmus, high myopia, and strabismus [1]. The disease can be caused by either faulty rod transduction or faulty transmission of the photoreceptor response to the inner retina, a distinction that is shown clearly by full-field electroretinography. All CSNB patients show a severely reduced or absent standard rod electroretinogram (ERG) (0.01 scotopic response), and most also have a strikingly electronegative combined rod-cone ERG (scotopic 3.0 response), with a strong a-wave but reduced b-wave. This type of CSNB was first described by Schubert and Bornschein [2] and may be accompanied by nystagmus, moderately high myopia, and strabismus [1]. Although the “complete” form of the disease results from faulty ON-pathway transmission between rods and bipolar cells, there are “incomplete” forms of this disease with lesser rod loss and variable cone (and OFF-pathway) involvement [2, 3].

In contrast, a small subgroup of CSNB patients shows a very different type of ERG. Instead of a “negative” combined rod-cone ERG, they show small responses with loss of the rod a-wave as well as the b-wave. This type of CSNB was first described by Riggs [4], later identified in a large French pedigree (Nougaret family) [5–7], and it represents a failure of rod transduction. These patients usually have normal vision in light and do not have nystagmus and strabismus and may or may not have myopia.

Whereas CSNB with a negative ERG is almost always inherited in a recessive (ar) or x-linked fashion, the Riggs-type of CSNB may be autosomal recessive (ar) or autosomal dominant (ad) [1]. To date, Riggs-type adCSNB has been found with mutations in* RHO* (which codes for rhodopsin) (MIM: 180380) [8–11], in* PDE6B* (which codes for the *β* subunit of phosphodiesterase) (MIM: 180072) [12, 13], and* GNAT1* (which codes for the *α* subunit of transducin) (MIM: 139330) [5, 14]. Riggs-type arCSNB has also been observed with defects in* GNAT1* [15] and* SLC24A1* (which codes for the solute carrier family 24 (sodium/potassium/calcium exchanger), member 1) (MIM: 603617) [16, 17]. All of these gene defects can also account for progressive rod-cone dystrophies [18–25].

Constitutive activation of these photoreceptor genes was proposed as the underlying pathogenic mechanism associated with most cases of Riggs-CSNB type of ERG [26–28]. The* GNAT1* mutations have been thought to interfere with GTP-binding but also with binding of the inhibitory PDE6*γ* subunit [14, 29]. In contrast, truncated, trafficking deficient or misfolded mutants of these genes were associated with progressive rod-cone dystrophy (RCD) [1, 24, 30].* GNAT1* defects are very rare in both CSNB and RCD, and to date only two mutations have been described causing adCSNB, one causing arCSNB and two other causing arRCD [5, 14, 15, 23, 24]. We recently saw a Chinese family with Riggs-type adCSNB whose gene testing revealed a new causative mutation in* GNAT1 *which appears to have a different mechanism of action compared to ineffective binding to GTP.

## 2. Materials and Methods

### 2.1. Clinical Studies

The patients were clinically investigated at the Byers Eye Institute at Stanford, having been referred to one of the authors (MFM). All studies were performed for clinical and not research indications. Examination included central fundus photography, wide-field photography and fundus autofluorescence (Optos), macular autofluorescence (FAF) (Heidelberg), spectral-density optical coherence tomography (OCT) (Zeiss, Cirrus), color vision testing (saturated and desaturated D-15 tests), full-field electroretinography (ERG), and visually evoked potentials (VEP) (Espion 3, Diagnosys LLC) in accordance with the International Society for Clinical Electrophysiology of Vision Standards [31].

### 2.2. DNA Extraction

Research procedures adhered to the tenets of the Declaration of Helsinki. Prior to genetic testing, written informed consent was obtained from each study participant. Blood samples of the affected father and daughter (3043.9190.16, I.1 and 17.9189.17 II.1, resp.) were collected and genomic DNA was extracted using a spin column method. DNA quality and quantity were assessed through gel electrophoresis and fluorometric analysis, respectively.

### 2.3. Targeted Next Generation Sequencing (NGS)

Initial DNA analysis was performed by a company (Blueprint Genetics, Helsinki, Finland). DNA was passed on an oligonucleotide-selective sequencing (OS-Seq) panel, covering the coding regions of 17 genes known to be associated with CSNB. Putative disease causing variants were validated by Sanger sequencing. In addition, copy number variation (CNV) analysis was performed. Deletions and duplications were detected from the targeted next generation sequencing (NGS) data using a proprietary bioinformatics pipeline, which processes aligned sequence reads by the company OS-Seq variant calling pipeline. Identified deletions and duplications were compared to their in-house curated and maintained database and public databases (Database of Genomic Variants (DGV)) [32] and DECIPHER (https://decipher.sanger.ac.uk/news) [33] to estimate the pathogenicity of the aberrations.

### 2.4. Sanger Sequencing

To validate the* GNAT1* variant identified by the company, a bidirectional Sanger sequencing was performed as previously reported [24, 34] using specific oligonucleotides in exons 2 and 3 of* GNAT1* (human* GNAT1* reference sequence: NM_144499.2: exon 2 forward: 5′-GGACTTAATTTGGATGGGGG-3′; and exon 3 reverse: 5′-GTCTGCCATGTGCATCAGC-3′) (Microsynth, Balgach, Switzerland).

### 2.5. Pathogenic Predictions Programs

To predict pathogenic mechanism, a software program (Alamut Visual 2.7-1, Interactive Biosoftware) was used, combining different programs such as Sorting Intolerant From Tolerant (SIFT, http://sift.jcvi.org/), [35], Polymorphism Phenotyping v2 (PolyPhen-2, http://genetics.bwh.harvard.edu/pph2/), [36], and Mutation Taster (http://www.mutationtaster.org/) [37]. This analysis also delivers frequencies in known databases such as Database of Single Nucleotide Polymorphisms (dbSNP, https://www.ncbi.nlm.nih.gov/snp), Exome Aggregation Consortium (ExAC, http://exac.broadinstitute.org/), and Exome Variants Server (EVS, http://evs.gs.washington.edu/EVS/). In addition, the presence of the variant in common databases was investigated using 1000 Genomes (http://www.1000genomes.org/) and gnomAD (http://gnomad.broadinstitute.org/). The Human Gene Mutation Database HGMD® Pro was consulted to investigate for known variants implicated in disease.

### 2.6. Domain and Three-Dimensional Structure of GNAT1 Predications

Specific domains of GNAT1 were predicted with respect to (1) the crystal structure of bovine transducin-*α* complexed with GTP*γ*S, (2) interaction studies between transducin-*α* and the inhibitory subunit of the subunit of phosphodiesterase-*γ* by photo-cross-linking approaches [38, 39], (3) prediction of a nuclear localization signal (NLS) (http://nls-mapper.iab.keio.ac.jp/cgi-bin/NLS_Mapper_y.cgi) [40], and (4) Uniprot (http://www.uniprot.org/uniprot/P11488) [41]. NLS sequence scores predict protein localization: proteins with a score of 8, 9, or 10 exclusively localize to the nucleus, with a score of 6 or 7 partially localized to the nucleus, with a score of 3, 4, or 5 localized to both nucleus and cytoplasm, and with a score of 1 or 2 localized to the cytoplasm (Tables 1 and 2). The three-dimensional structure of the wild-type GNAT1 form, the two previously reported adCSNB mutants (p.Gly38Asp and p.Gln200Glu) [5, 14], the previously reported arCSNB mutant (p.Asp129Gly) [15], and the new adCNSB mutant reported herein (p.Ile52Asn) were predicted with wild-type and mutant protein sequences using human GNAT1 protein sequence reference: NP_653082.1 (Iterative Threading ASSEmbly Refinement, I-TASSER, https://zhanglab.ccmb.med.umich.edu/I-TASSER/ [42]; TM-Align, https://zhanglab.ccmb.med.umich.edu/TM-align/) [43]. The PyMOL Molecular Graphics System, Version 1.7.x Schrödinger, LLC, was used to model GNAT1 interactions with GTP/GTP*γ*S.

### 2.7. Immunolocalization Studies in COS1 Cells Overexpressing Wild-Type and Mutated GNAT1

The DNA coding sequence without the stop codon and HindIII and XbaI linkers of the wild-type and mutated human* GNAT1* were synthesized in an optimized way and cloned in a mammalian expression vector pBudCE4.1 (Thermo Fisher, Villebon-sur-Yvette; GeneCust, Dudelange, Luxembourg). This vector contains a C-terminal myc tag, which allows detecting the protein by immunolocalization with an anti-myc antibody in case the antibody directed against the endogenous protein is not working. Transient transfection studies were performed in COS-1 cells similarly as previously described [44]. To detect the protein cells were stained with either mouse anti-GNAT1 antibody (sc136143, Santa Cruz Biotechnology, CliniSciences, Nanterre, France) or anti-myc antibody (11667149001, Roche, Basel, Switzerland) and secondary anti-mouse Cy3 mouse antibody (711-165-150, Jackson ImmunoResearch Laboratories, Baltimore, MD, USA). Subsequently cells were stained with DAPI (40,6-diamidino-2-phenylindole) (AAT Bioquest, Sunnyvale, Etats Unis) and mounted in mounting medium (Fluoromount-G, Southern Biotech, Birmingham, AL, USA) using coverslips. Cell preparations were visualized with standard fluorescence microscopy (DM6000, Leica, Wetzlar, Germany) at a 60x magnification.

## 3. Results

### 3.1. Clinical Findings

**Figure 1:**
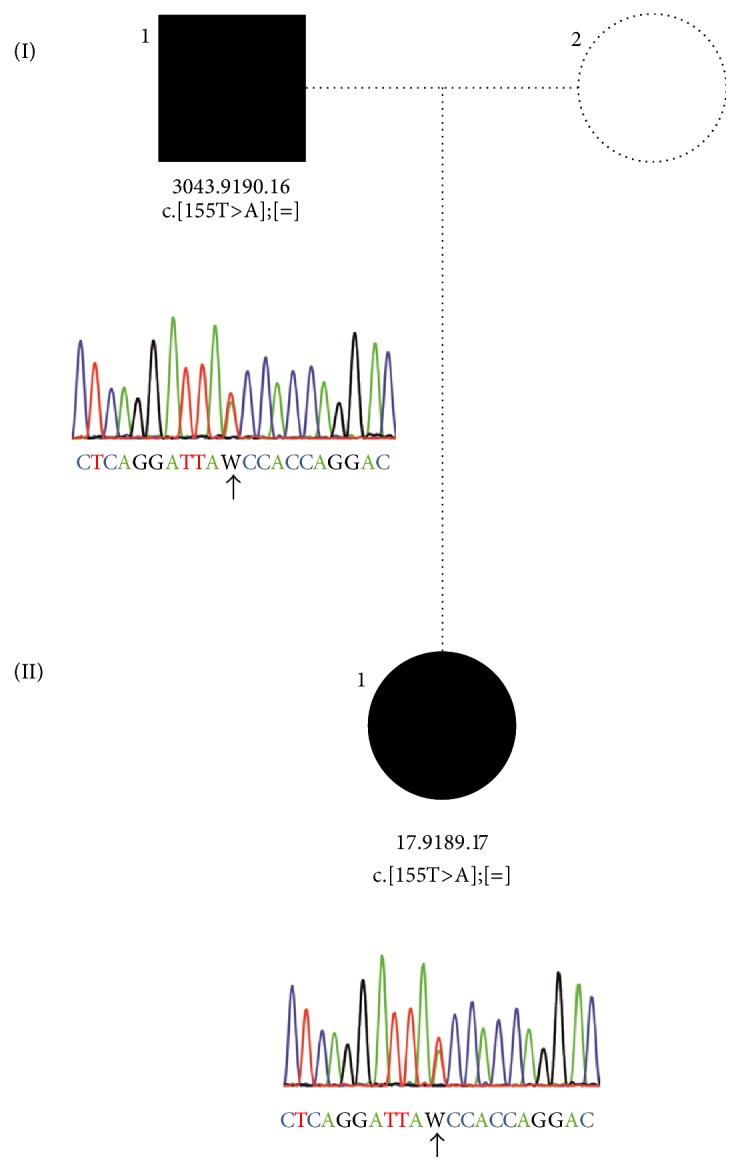
Validation and cosegregation of* GNAT1* variant in the family with adCSNB. The pedigree and respective electropherograms of each tested family member are depicted. The family is composed of an affected father (3043.9190.16; I.1) and one affected daughter (17.9189.17; II.1). The missense variant c.155T>A p.Ile52Asn in* GNAT1* was found at heterozygous state in the affected father and daughter. Females and males are depicted by circles and squares, respectively. Filled and unfilled symbols indicate affected and unaffected status, respectively. The arrow indicates the nucleotide position 155 heterozygously changed in the father and daughter.

**Figure 2:**
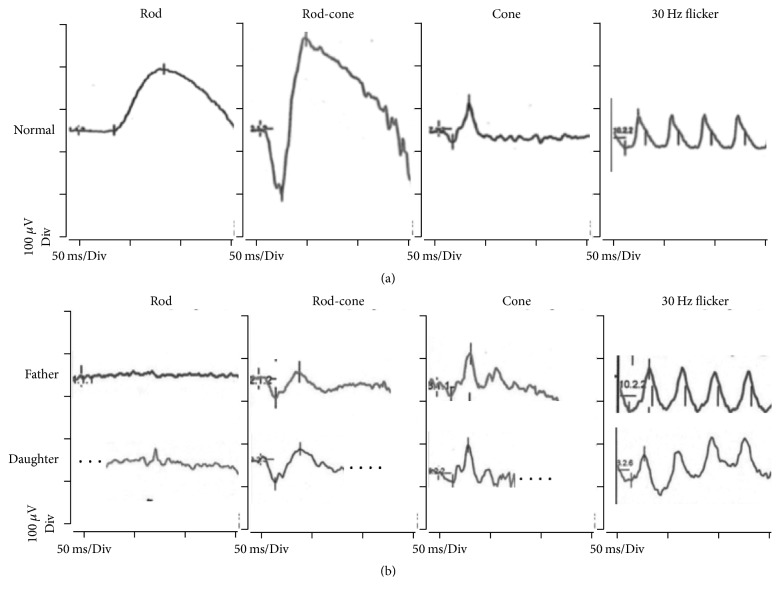
Electroretinograms of the family with adCSNB show Riggs phenotype. Representative responses are shown (a) from a normal eye and (b) from father and daughter. Note, total absence of the rod responses (scotopic 0.01 stimuli) and rod-cone responses showing only a waveform similar to the cone signals (scotopic 3.0 stimuli). The cone responses (photopic 3.0 stimuli) were normal, as were the times-to-peak of the flicker responses. Dotted lines replace movement artifact.

The patients were a Chinese father and daughter (from Hong Kong) (Figure 1), aged 42 and 20 years at the time of examination. Both have had life-long severe night blindness. The daughter is an only child, and the father has two unaffected brothers; he is uncertain about symptoms in his parents. Both have been in good health except that both have postural orthostatic tachycardia syndrome (POTS) (MIM: 604715). Color testing of the daughter was normal with both saturated and desaturated D-15 panels. Both corrected to 20/25 in OD and 20/20 in OS; the father was roughly −10 D myopic and the daughter −3 D. There was no nystagmus or strabismus. Retinal exam was unremarkable except for tilted myopic discs and some myopic depigmentation particularly in the peripapillary area. Wide-field and central fundus autofluorescence images showed no retinal degeneration, and the maculae were normal on SD-OCT. The father had been told that his problem was optic nerve disease and was referred for a VEP as well as an ERG. The VEP was normal. The ERG in both subjects showed typical findings of Riggs-type CSNB (Figure 2) with no rod response to a weak (0.01) or strong (3.0) scotopic flash, and the combined rod-cone 3.0 scotopic response looked like a cone b-wave. The photopic 3.0 cone and flicker responses were essentially normal.

### 3.2. Genetic Findings

The commercial targeted OS-Seq combined with NGS analysis revealed a heterozygous deletion in* LRIT3* (c.1551_1552del p.Leu518Valfs*∗*54) in the daughter, which is predicted to lead to a frameshift and premature truncation of the protein. More strikingly, the patient showed a novel mutation in* GNAT1* (c.155T>A p.Ile52Asn), which was confirmed by Sanger sequencing in the father and daughter and which is indeed the most likely cause of the phenotype in both (Figure 1). It is predicted to be pathogenic by PolyPhen-2, SIFT, and Mutation Taster; it has never been reported in patients with other retinal disorders nor in the general population and affects an amino acid residue which is highly conserved (99 species show Ile and 1 Leu).

### 3.3. Pathogenic Mechanism

To better understand the pathogenic mechanism of the* GNAT1* mutation identified herein leading to adCSNB (p.Ile52Asn in the context of the other mutations leading to adCSNB (p.Gly38Asp and p.Gln200Glu) and to arCSNB (p.Asp129Glu)), we investigated which domains of each mutant were predicted to be affected and performed immunolocalization studies and 3D-modelling of all mutations implicated so far in CSNB.

### 3.4. Domains Affected by Mutations in GNAT1

The previously identified adCSNB mutations (p.Gly38Asp and p.Gln200Glu) were predicted to be located in a GTP-binding domain, while the previously identified arCSNB mutation (p.Asp129Glu) was found in an unknown domain. Interestingly, one of the previously described adCSNB mutations, p.Gly38Asp, and the novel p.Ile52Asn are located in a region, which may represent a nuclear localization signal (NLS) (score 5,6) and thus these mutations may affect the subcellular localization of the mutant GNAT1 proteins (Tables 1 and 2).

### 3.5. GNAT1 CSNB Mutations Do Not Alter the Subcellular Localization

**Figure 3:**
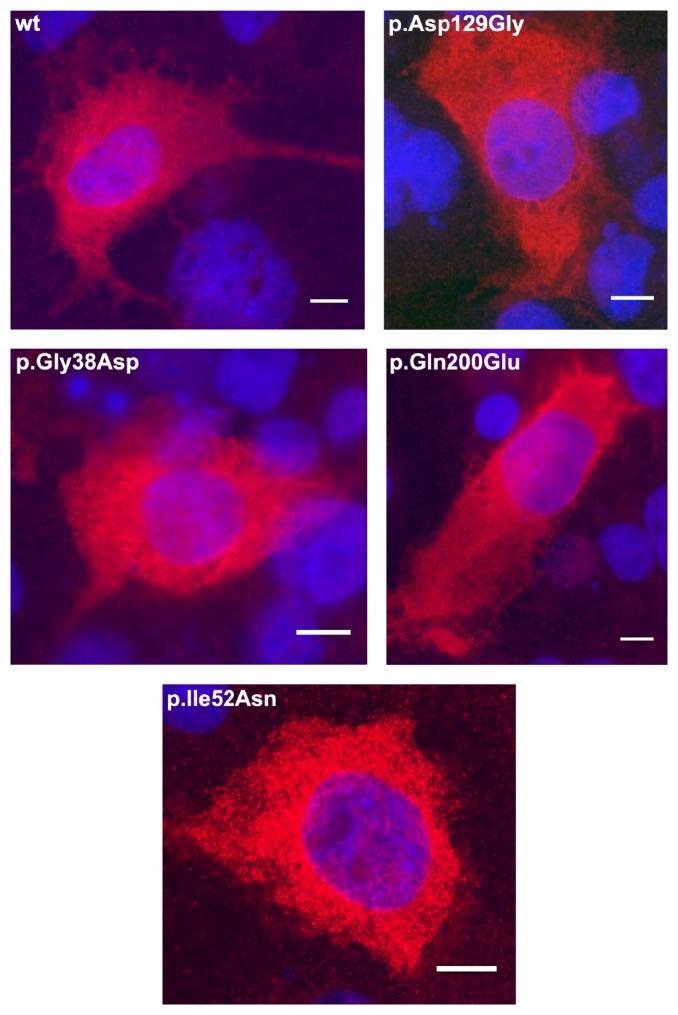
Localization of wild-type GNAT1 and mutated variants overexpressed in COS-1 cells. The wild-type and mutant GNAT1 protein was detected by a mouse GNAT1 antibody (red). The nuclei were stained with DAPI (blue).

To exclude that mislocalization of mutant protein is the underlying pathogenic mechanism leading to CSNB, the wild-type and all 4 mutants harboring missense mutations were studied using myc-tagged constructs overexpressed in COS-1 cells. However, no differences in the subcellular staining of GNAT1 and the different CSNB mutants were observed using anti-GNAT1 or anti-myc antibodies. In all different cell preparations, many cells showed (1) solely cytosolic, (2) cytosolic and partial nuclear staining (Figure 3, shown for anti-GNAT1 antibody), and some (3) cytosolic staining with a sharp ring around the nucleus. Membrane localization with the antibodies used could not be detected using life cell staining. Together these findings indicated that the mutations implicated in CSNB do not alter the subcellular localization of GNAT1.

### 3.6. GNAT1 CSNB Mutations Alter Differently the 3D-Structure

**Figure 4:**
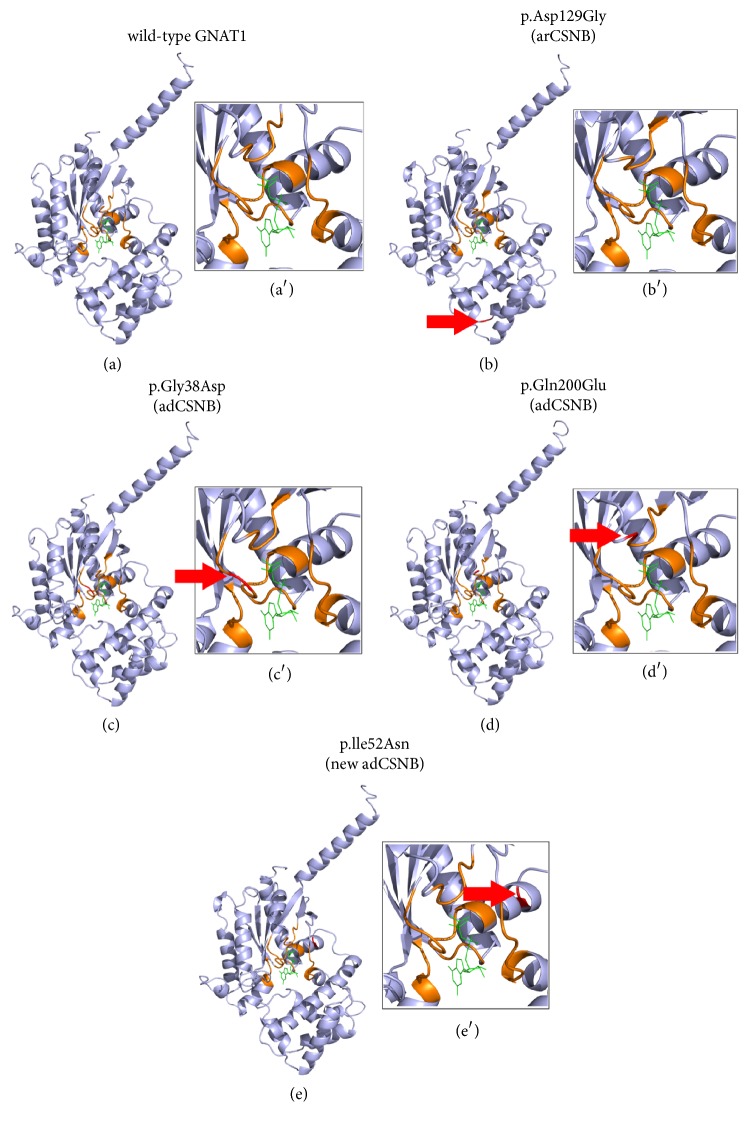
3D-model of normal and mutant GNAT1 interacting with GTP*γ*S. The three-dimensional structure of (a) the GNAT1 wild-type; (b) the previously reported autosomal recessive CSNB mutant, p. Asp129Gly [15]; and the three autosomal dominant CSNB mutants: (c) p.Gly38Asp [5]; (d) p.Gln200Glu [14]; and (e) p.Ile52Asn. All GNAT1 variants are colored red and marked with a red arrow. The GTP-binding regions are colored in orange and GTP is colored in green. The novel autosomal dominant CSNB variant, p.Ile52Asn, does not affect the GTP-binding domain (in orange), while the two autosomal dominant CSNB variants, previously reported, p.Gly38Asp and p.Gln200Glu, are located in the GTP-binding domain (orange) and thus are predicted to affect the interaction with GTP*γ*S (in green). A close-up of GTP-binding site was done for each construction, normal (a′) and GNAT1 mutants (b′, c′, d′, and e′).

3D modelling (Figure 4) confirmed that the two previously reported adCSNB variants, p.Gly38Asp and p.Gln200Glu (Figures 4(c, c′) and 4(d, d′), resp., colored in red and red arrow) are localized in the GTP-binding site (orange region) surrounding GTP (green) (Figure 4, Tables 1 and 2), as previously described [14, 29, 45, 46]. The amino acids affected in the arCSNB case (p.Asp129Gly) (Figure 4(b) colored in red and red arrow and not present in 4(b′)) and in the novel adCNSB case identified herein (p.Ile52Asn) (Figure 4(e) and 4(e′), red and red arrow) are absent of the GTP-binding binding domain (orange). Although the p.(Ile52Asn) variant (in red, red arrow) is localized in the *α*-helix whom C-terminal interacts with GTP/GDP (green), the position 52 is most likely too far away to impact on the three-dimensional structure of the GTP-binding site (Figure 4).

## 4. Discussion

Although the* LRIT3* variant was not reported in common databases and is predicted to be disease causing, it cannot be responsible for the Riggs-form of CSNB observed in this family as the father does not show this variant. Furthermore, CSNB caused by* LRIT3* mutations is inherited in an autosomal recessive manner and shows the Schubert-Bornschein rather than Riggs ERG phenotype [47]. However, the patients also revealed that a novel* GNAT1* variant (p.Ile52Asn), which cosegregated with the phenotype, was predicted to be pathogenic and was never reported in the general population. The mutations in* GNAT1* adCSNB reported previously were p.Gly38Asp in the Nougaret family [5, 48] and p.Gln200Glu [14] in another family. Thus, our findings reveal a third* GNAT1* mutation causing adCSNB. The ERG phenotype fits the Riggs-form of CSNB with absent rod photoreceptor responses, while the cone system is largely preserved. Riggs-type CSNB does not typically show reduced visual acuity, nystagmus, or strabismus, and that was true of our patients. Myopia is not a routine concomitant either (as it is with many patients having Schubert-Bornschein type of CSNB), and our patients had only mildly myopic fundi with refractive errors from −3 D to −10 D.

It is intriguing that both father and daughter also have postural orthostatic tachycardia syndrome (POTS) and poorly understood autonomic dysfunction. It is most often an acquired condition, or at least not congenital, associated with stress or with metabolic changes such as pregnancy or menstruation in woman pattern. It is intriguing to speculate on whether the condition might involve genetic overlap with the* GNAT1* defect in this family. Recently, a* SLC6A2 *mutation in an autosomal dominant pedigree was associated with this phenotype [49], a finding which was further validated in a mouse model carrying the same mutation [50]. Genetically,* SLC6A2* is not related to* GNAT1*. It might be interesting to see if our family has as well an independent mutation in* SLC6A2*, which was however not the purpose of this work. In addition, we cannot exclude that this is due to another gene defect cosegregating with this phenotype, which may be independent of the CSNB-phenotype. Whole exome or whole genome sequencing could be done to verify these different hypotheses.

Although few Riggs-CSNB cases have been published, a number of studies have investigated the pathogenic mechanism, perhaps because the genes mutated in these cases can lead either to RCD or CSNB [1]. The GNAT1 abnormalities leading to arRCD have been truncating mutations that affect the C-terminal region and the predicted 3D structure seems only marginally affected [23, 24]. It has been suggested that RCD is due to inability of transducin to bind GTP/GDP and activated RHO rather than a degraded mutant protein.

In contrast, missense mutations in* GNAT1* have been associated with adCSNB or arCSNB [24]. Our results suggest that mislocalization is not the underlying mechanism leading to adCSNB or arCSNB, as this was not observed with any of the known mutations. Our studies and previous findings showed that the two previously reported adCSNB variants, p.Gly38Asp and p.Gln200Glu, are localized in the GTP-binding site (orange region) [14, 29, 45, 46]. Such a position is typically predicated to lead to constitutive activation. But, functional* in vitro* assays and transgenic mouse studies for the p.Gly38Asp mutant revealed the inability of the activated mutant GNAT1 to bind to the inhibitory *γ* subunit of PDE6 and activate PDE6 [29]. Interestingly, domain predictions did not forecast that the p.Gly38Asp affect the binding domain of *γ* subunit of PDE6. However, constitutive activation is still suggested for the p.Gln200Glu variant identified in human adCSNB [14]. Therefore, although both previously described adCSNB mutations in GNAT1 were predicted to lead to constitutive activation, this seems to be true only for the p.Gln200Glu GNAT1 variant.

Unlike these previously described adCSNB GNAT1 mutations, the novel variant identified in our family, p.Ile52Asn, is absent from the GTP-binding interacting domain. Thus, it may involve another pathogenic mechanism, presently unknown. The underlying pathogenic mechanism associated with the arCSNB GNAT1 mutant, p.Asp129Gly, also remains unclear [15] as it also localizes outside the binding domain. This mutation is predicted to modify hydrogen bonding to the surrounding amino acid and may induce structural abnormalities important for the proper function of the protein. Functional analyses using* in vitro* or to be developed transgene* in vivo* models could shed light on the different pathogenic mechanisms underlying* GNAT1 *mutations.

## 5. Conclusion

There are several implications to be derived from this work. We describe a new genetic pedigree for Riggs-type adCSNB that confirms the clinical distinctions between transduction-modulated CSNB and transmission-modulated CSNB. We note an unexplained overlap symptomatically with the POTS syndrome. We describe a third GNAT1 abnormality which can cause adCSNB. Immunolocalization studies show that this p.Ile52Asn mutation localizes within cells identically (primarily cytosolic) as other CSNB causing GNAT1 mutants. However, structural analysis shows that whereas the other adCSNB GNAT1 mutations localize at the GTP-binding site, p.Ile52Asn does not (being some distance further away). This suggests that mechanisms other than interference with GTP-binding may be the cause of disease in this new mutation.

## Figures and Tables

**Table 1:** Domains of GNAT1 and mutations leading to autosomal dominant (ad) and autosomal recessive (ar) congenital stationary night blindness (CSNB) or ar rod-cone dystrophy (RCD).

Domains	Amino acids
*βγ*-Transducin binding site	1–23
Nuclear localization signal	21–52
GTP/GDP binding sites	36–43, 171–177, 196–200, 265–268, and 321–323
Unknown region	129
Magnesium binding site	43, 177
PDE6*γ* inhibitory binding site	306–310
Activated RHO binding site	311–328 and 340–350

NLS = nuclear localization signal.

**Table 2:** Domains affected in patients with *GNAT1* leading to autosomal dominant (ad) and autosomal recessive (ar) congenital stationary night blindness (CSNB) or ar rod-cone dystrophy (RCD).

Mutation (aa)	Mode of inheritance and phenotype	Domains affected	Reference
p.Gly38Asp	adCSNB	NLS, GTP/GDP binding sites	[5]
p.Ile52Asn	adCSNB	NLS	Reported herein
p.Gln200Glu	adCSNB	GTP/GDP binding sites	[5, 14]
p.Asp129Gly	arCSNB	unknown	[15]
p.Gln302*∗*	arRCD	Truncates: GTP/GDP binding sites, PDE6*γ* inhibitory binding site and activated RHO binding site	[23]
p.Cys321*∗*	arRCD	Truncates: GTP/GDP binding sites and activated RHO binding site	[24]

NLS = nuclear localization signal.
